# A Two-Dimensional Numerical Investigation of Transport of Malaria-Infected Red Blood Cells in Stenotic Microchannels

**DOI:** 10.1155/2016/1801403

**Published:** 2016-12-26

**Authors:** Tong Wang, Yong Tao, Uwitije Rongin, Zhongwen Xing

**Affiliations:** ^1^Department of Mathematics, Nanjing University of Aeronautics and Astronautics, Nanjing 210016, China; ^2^School of Electronic Science and Engineering, Nanjing University, Nanjing 210093, China

## Abstract

The malaria-infected red blood cells experience a significant decrease in cell deformability and increase in cell membrane adhesion. Blood hemodynamics in microvessels is significantly affected by the alteration of the mechanical property as well as the aggregation of parasitized red blood cells. In this study, we aim to numerically study the connection between cell-level mechanobiological properties of human red blood cells and related malaria disease state by investigating the transport of multiple red blood cell aggregates passing through microchannels with symmetric stenosis. Effects of stenosis magnitude, aggregation strength, and cell deformability on cell rheology and flow characteristics were studied by a two-dimensional model using the fictitious domain-immersed boundary method. The results indicated that the motion and dissociation of red blood cell aggregates were influenced by these factors and the flow resistance increases with the increase of aggregating strength and cell stiffness. Further, the roughness of the velocity profile was enhanced by cell aggregation, which considerably affected the blood flow characteristics. The study may assist us in understanding cellular-level mechanisms in disease development.

## 1. Introduction

Malaria infects 350–500 million people and kills more than two millions every year, mostly in Africa and other underdeveloped countries. In most of the malaria cases, red blood cells (RBCs) are parasitized by* Plasmodium falciparum* (*P*-falciparum), a protozoan parasite transmitted by mosquitos. When the RBCs are parasitized by *P*-falciparum, two critical effects have been observed in the infected cells, namely, the reduced deformability of the cell and increased adhesion of the RBCs to vessel endothelium and other blood cells [1]. Thus, the parasitized RBCs become stiffer (could be tenfold harder than healthy RBCs [2]) and tend to form aggregates in blood. Blood flow may be substantially affected by the change of structural and mechanical properties of the malaria-infected RBCs.

Under healthy physiological conditions, red blood cells move individually with blood plasma or form temporal aggregates and they can undergo severe, reversible, large elastic deformation in blood flow. Although the formation of red blood cell aggregates could be due to other factors such as the decrease of the shear rate, increase of the hematocrit, and variation of the viscosity of the suspending plasma in the blood vessel, the malaria-infected RBCs tend to form firm and irreversible aggregates. Severe aggregation of RBCs may decrease the surface area of cells contacting with the blood plasma. It also causes pressure and shear stress change in the region of the aggregation. These aggregates get stuck in the small blood vessels, blocking individual RBC from passing, thus decreasing the amount of oxygen and the nutrition transferred in human body.

Experimental measurements have been done on dynamic or rheological properties and behaviors of parasitized RBCs [3–10]. In the experiments, microfluidic channels have been employed to probe the deformability of healthy and malaria-infected red blood cells. The hardening of the cell and the blockage of the channel can be qualitatively studied. However, a major difficulty in carrying out experimental investigations on malaria-infected RBCs is the complexity of the microvessels. Moreover, experimental techniques are in general not fully capable of illustrating cellular-level rheological behaviors of RBC aggregates. Because of the relatively large number of red blood cells, cell-to-cell interactions and local rheological properties were unable to identify. Therefore, numerical simulations have been utilized as an alternative tool to study the rheology of parasitized RBCs in blood flow [11–15]. Numerical models that have been used for interpretation and prediction of mechanical properties and dynamic behavior of RBCs in malaria include dissipative particle dynamics (DPD) [13, 15], smoothed particle hydrodynamics (SPH) [14], lattice Boltzmann method (LBM) [16], and others [17]. Two or multiple RBCs are considered by numerical simulations to probe their interactions, for example, their aggregation and disaggregation in blood flow [18–22].

However, very few investigations on malaria-infected RBCs have been conducted in stenotic vessels and their impact on hemodynamics in stenotic vessels is not well understood. In particular, there is a lack of studies considering cell-to-cell interactions at microscopic scale. This paper aims to provide a qualitative analysis on the dynamics of malaria-infected RBCs in stenotic microvessels. Since the malaria-infected RBC gradually loses its deformability and develops adhesiveness through the three stages of infection ring, trophozoite (early trophozoite, late trophozoite), and schizont, this study considers the ring and trophozoite stages in which the cells become stiffer and more adhesive while the shape of the RBCs is not significantly modified. The RBC was simulated by a spring model and the hardening of the parasitized cell was mimicked by varying the membrane constants. The malaria-infected RBCs were assumed existing as aggregates with firm or loose adhesion. By using fictitious domain-immersed boundary formalism in two dimensions, we simulated the transit of RBC aggregates through a prototypical channel with a contraction. By studying the motion and dissociation of RBC aggregates and flow characteristics, we delineated the contribution to hemodynamics from each of the three factors, hardening of the membrane, aggregation strength, and stenosis magnitude.

## 2. Methods

We considered a two-dimensional microvessel with a symmetric stenosis and employed numerical simulations to study the rheological behavior of RBC aggregates in the blood flow. In this study, blood was assumed to be a suspension of RBCs in an incompressible, Newtonian fluid with constant density and viscosity. In order to simulate the blood flow and fluid-cell interactions in this irregular-shaped domain, the fictitious domain method was combined with the immersed boundary method and the RBCs have been modeled by the spring model.

### 2.1. Red Blood Cell Model

Two types of RBC model are widely used currently, namely, elastic membrane models [19, 23] and spring models [20, 24–27]. In this paper, we adopted the spring model introduced in [24, 28] and modeled individual RBC as cytoplasm enclosed by a membrane represented by a finite number of membrane particles connected by springs. The springs of stretch/compression and bending modulus change its length and the bending angle between two neighboring springs under external force. The elastic moduli are affected in disease conditions [29, 30]. Evidence shows that malaria-infected RBC has increased stiffness, which is closely related to the cell bending modulus. In this paper, we modeled malaria-infected RBC with different deformability by varying the bending constant of the spring. The shape of the RBCs was chosen with the reduced area *s* ^*∗*^ = 0.481.

### 2.2. Aggregation Kinetics

The hardening of the RBCs is due to the parasite of the *P*-falciparum. However, the stickiness of the cell surface and the mechanism of the aggregation still remain disputable. Two different theoretical models, namely, the bridging model [18, 31] and the depletion model [32], coexist nowadays. Although they are distinct in the cause of the aggregating force, both of them predict the force being attractive at far and repulsive at near distance. Because the investigation of the mechanism of aggregation is not within the scope of this research, we chose a simple model [20] in which aggregation of RBCs was achieved by introducing an aggregating force existing in a neighborhood of membrane particles and the aggregating force was derived from *F* = −∂*E*/∂*r* with the Morse-type potential *E* as

(1) $$\begin{array}{c} E=D_{e}\left(\;e^{\left(2\beta \left(r_{0}-r\right)\right)}-2e^{\left(\beta \left(r_{0}-r\right)\right)}\;\right), \end{array}$$

where *D*_*e*_ is the energy constant corresponding to the density of the aggregating particles; *β* is the scaling factor which relates to the thickness of the depletion layer or interactive distance; *r*_0_ is the reference distance or zero-force distance.

**Figure 1:**
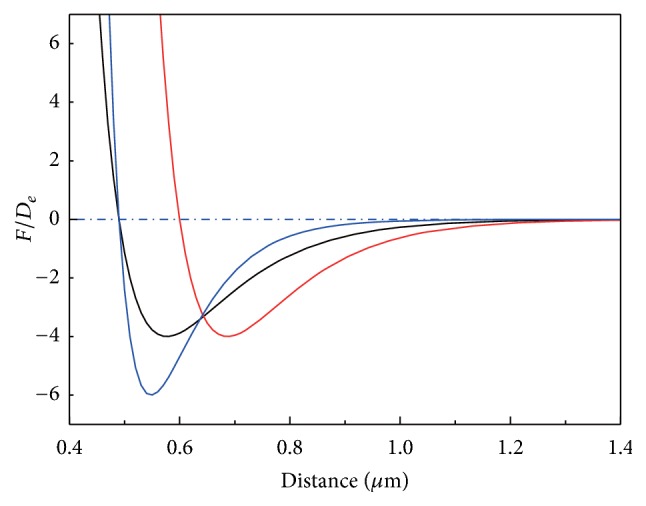
Dependence of aggregation force on distance for various parameters: black line: *β* = 80 *μ*m^−1^ and *r*_0_ = 0.49 *μ*m; red line: *β* = 80 *μ*m^−1^ and *r*_0_ = 0.6 *μ*m; blue line: *β* = 120 *μ*m^−1^ and *r*_0_ = 0.49 *μ*m.

The scaling aggregation force (*F*/*D*_*e*_) as a function of separation distance for different parameters is shown in Figure 1. When the value of the scaling force is negative, the force is attractive; when the value of the force is positive, the force is repulsive. The force decreases almost to zero at far enough distance. Aggregation could be increased either by increasing *D*_*e*_ or by decreasing *β*. On the other hand, decreasing in *β* and increasing in *r*_0_ may raise the depletion thickness and the equilibrium distance of the RBCs in aggregates.

### 2.3. The Fictitious Domain-Immersed Boundary Scheme

The flow region we studied was a stenotic microchannel for which the regular structured mesh was not applicable at the boundary of the region. Thus, we adopted the fictitious domain method because in this method the irregular-shaped domain is extended to regular shape so that simple structured mesh instead of unstructured mesh can be used, which substantially reduces computational complexity of the algorithm. The fictitious domain method and its applications to fluid flow problems have been extensively described [33, 34]. To employ the fictitious domain method, the flow region *Ω*_*f*_ was embedded in a rectangular domain denoted by *Ω*. Then the fluid flow containing RBCs was solved in the bigger domain *Ω*, and the no-flow condition in the solid region was treated as constraints. Therefore, the governing equations for the modeled problem were the following extended Navier-Stokes equations:

(2) ρ∂u∂t+u·∇u=−∇p+μΔu+f,in Ωf,∇·u=0,in Ωf,u=0,in Ω∖Ωf,

where **u**(**x**, *t*) and *p* are the fluid velocity and pressure anywhere in the flow; *ρ* is the fluid density; *μ* is the fluid viscosity. The boundary conditions were such that, on ∂*Ω*_*f*_, a no-slip condition was applied and, at the inlet and outlet of the channel, a periodic flow condition was enforced. A detailed description of the solution method of (2) can be found elsewhere [33, 34]. In this study, the fluid-cell interaction was dealt with by the immersed boundary method developed by Peskin et al. [20, 35].

## 3. Results and Discussion

We studied the hydrodynamic behavior of multiple red blood cell aggregates in a horizontal channel filled with Newtonian fluid with a symmetric stenosis at the central part of the vessel. The blood plasma density and plasma viscosity were presumed fixed values. We performed a series of simulations to study RBC deformation, flow field, and cell-cell interactions as the aggregates traversed the stenotic vessel. The fluid flow was generated from left to right by a constant pressure drop. A stream of flow approaching the stenosis contracted to a high speed flow at the throat of the stenosis. Afterwards, the flow was allowed to develop fully along the straight rectangular channel. At the meantime, prelocated multiple RBC aggregates flowed with the fluid in the channel. The parameters used are given in Table 1. For the parameter used in this study, larger spring constant for RBC membrane and higher aggregation energy constant correspond to malaria-infected RBCs at more severe infection stages.

### 3.1. Aggregation of Red Blood Cells

**Figure 2:**
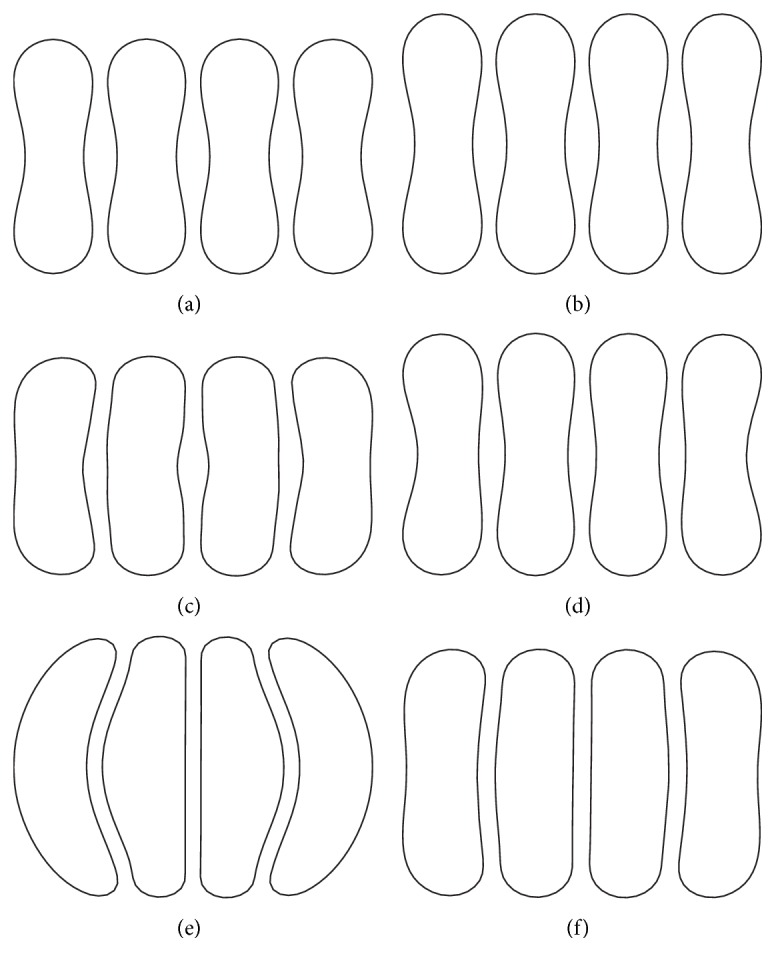
Equilibrium configuration of RBC aggregates for different simulation parameters: (a) *D*_*e*_ = 0.01 *μ*J/m^2^ and *k*_*b*_ = 3.0 × 10^−13^ Nm; (b) *D*_*e*_ = 0.01 *μ*J/m^2^ and *k*_*b*_ = 3.0 × 10^−12^ Nm; (c) *D*_*e*_ = 0.1 *μ*J/m^2^ and *k*_*b*_ = 3.0 × 10^−13^ Nm; (d) *D*_*e*_ = 0.1 *μ*J/m^2^ and *k*_*b*_ = 3.0 × 10^−12^ Nm; (e) *D*_*e*_ = 1.0 *μ*J/m^2^ and *k*_*b*_ = 3.0 × 10^−13^ Nm; and (f) *D*_*e*_ = 1.0 *μ*J/m^2^ and *k*_*b*_ = 3.0 × 10^−12^ Nm.

Aggregates of four cells used in the simulations were formed in static plasma before putting them in the flow channels. The red blood cells were placed face-to-face in static plasma initially. The center-to-center distance was close enough in order for the aggregation force to take effect. When the simulation starts, red blood cells approached each other and reached an equilibrium configuration. The red blood cells were modeled by two membrane constants for two different stiffness and three aggregation strengths for different adhesiveness. Therefore, six different configurations were obtained and they are shown in Figure 2. The configuration in Figure 2(a) corresponds to healthy RBC. The configurations in Figures 2(b)–2(d) may correspond to the ring stage and early trophozoite stage infection, while Figures 2(e) and 2(f) are assumed to correspond to the late stage of trophozoite in which both the stiffness and stickiness of the cell membrane increase considerably.

### 3.2. Motion and Dissociation of Aggregates

**Figure 3:**
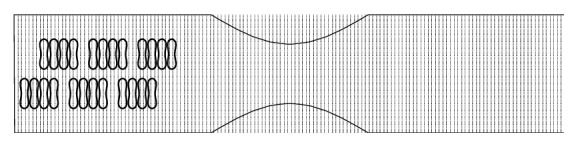
Schematic illustration of initial configuration of RBC aggregates in the blood vessel at the beginning of the simulations.

The microvessel in this study is a 140 *μ*m long, 30 *μ*m wide two-dimensional channel with symmetric stenosis formed at the central location. The geometry of the fluid domain and the initial placement of the RBC aggregates are illustrated in Figure 3. Blood flow in the stenotic vessel was driven by a constant pressure gradient imposed at the inlet and the outlet. The pressure gradient was determined such that the maximum flow velocity was about 20 cm/s (a typical value in arterioles [36]).

#### 3.2.1. In the Channel with 40% Stenosis

**Figure 4:**
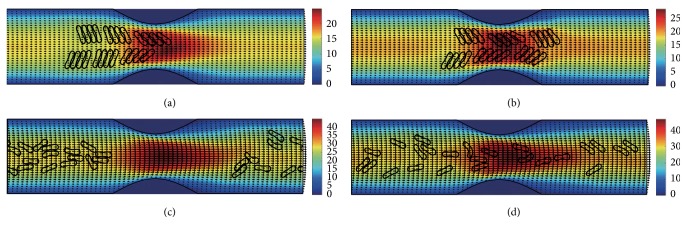
Motion of red blood cell aggregates (configuration in Figure 2(a)) in the microchannel with 40% stenosis at time instants (a) *t* = 0.35 ms; (b) *t* = 0.45 ms; (c) *t* = 1.34 ms; (d) *t* = 4.80 ms.

**Figure 5 fig5:**
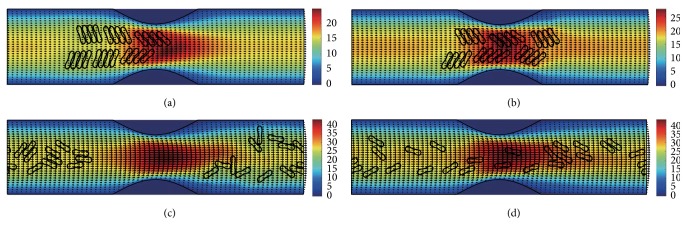
Motion of red blood cell aggregates (configuration in Figure 2(b)) in the microchannel with 40% stenosis at time instants (a) *t* = 0.35 ms; (b) *t* = 0.46 ms; (c) *t* = 1.35 ms; (d) *t* = 4.80 ms.

**Figure 6 fig6:**
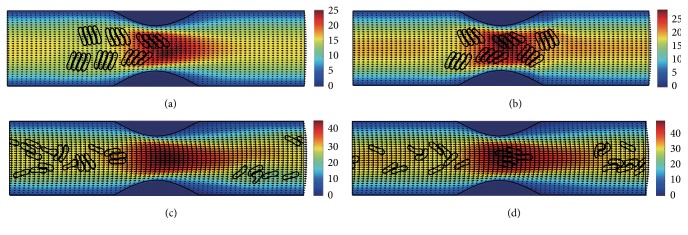
Motion of red blood cell aggregates (configuration in Figure 2(c)) in the microchannel with 40% stenosis at time instants (a) *t* = 0.35 ms; (b) *t* = 0.46 ms; (c) *t* = 1.37 ms; (d) *t* = 4.80 ms.

**Figure 7 fig7:**
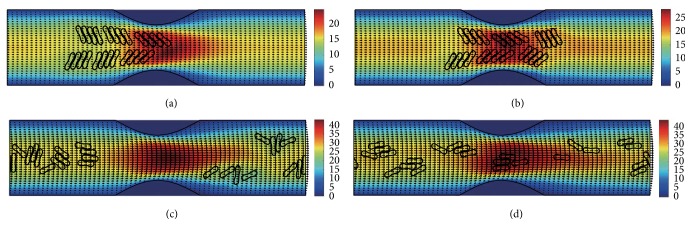
Motion of red blood cell aggregates (configuration in Figure 2(d)) in the microchannel with 40% stenosis at time instants (a) *t* = 0.35 ms; (b) *t* = 0.46 ms; (c) *t* = 1.36 ms; (d) *t* = 4.80 ms.

**Figure 8 fig8:**
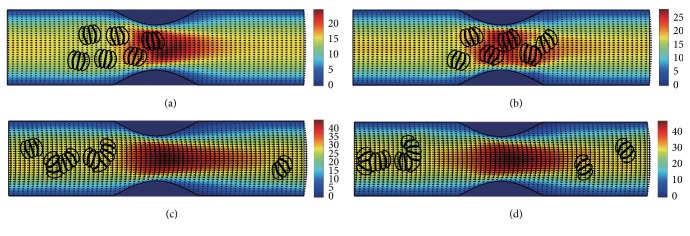
Motion of red blood cell aggregates (configuration in Figure 2(e)) in the microchannel with 40% stenosis at time instants (a) *t* = 0.35 ms; (b) *t* = 0.46 ms; (c) *t* = 1.39 ms; (d) *t* = 4.80 ms.

**Figure 9:**
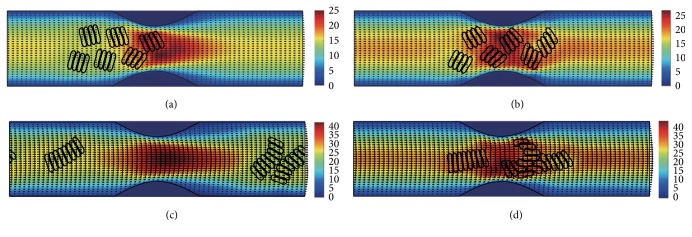
Motion of red blood cell aggregates (configuration in Figure 2(f)) in the microchannel with 40% stenosis at time instants (a) *t* = 0.35 ms; (b) *t* = 0.46 ms; (c) *t* = 1.37 ms; (d) *t* = 4.80 ms.

The motion and dissociation of 6 RBC aggregates in a 40% stenosis microvessel have been simulated. The results for two membrane bending constants and three aggregating strengths are shown in Figures 4 [Fig fig5] [Fig fig6] [Fig fig7] [Fig fig8]–9 for the initial configurations in Figure 2. The snapshots are for four time instants with the second subfigure showing the instant when the mean location of all cells was at the throat of the stenosis. When the RBCs are softer (Figures 4, 6, and 8), they experienced more deformation in the flow even after the dissociation. The aggregates with weak aggregating strength (Figures 4 and 5) were dissociated easily by the hydrodynamic force and the RBCs existed as dispersed individual cells after long enough elapsed time. The aggregates with mild aggregating strength (Figures 6 and 7) were partially disaggregated from their initial configurations. Smaller aggregates of two to three cells were observed in the simulation. However, when the aggregating strength was strong (Figures 8 and 9), the hydrodynamic force was unable to disaggregate the RBC aggregates. Aggregates underwent some deformation but however kept their initial configuration even at the stenosis. Because the velocity of the flow at the throat of the stenosis increased significantly due to the contraction, the aggregates lag behind which caught up the upstream ones. Eventually, agglomeration and rouleaux of RBCs of bigger size were formed by the aggregating force. In addition, more disturbance of the flow field around the aggregates or the cells has been noted.

#### 3.2.2. In the Channel with 50% Stenosis

**Figure 10:**
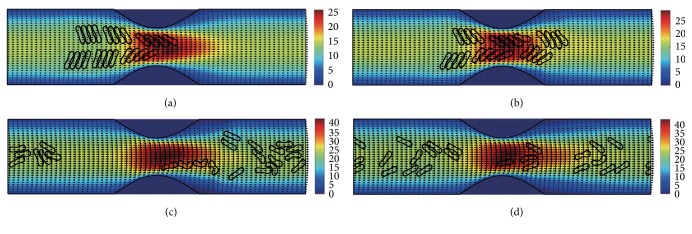
Motion of red blood cell aggregates (configuration in Figure 2(a)) in the microchannel with 50% stenosis at time instants (a) *t* = 0.37 ms; (b) *t* = 0.47 ms; (c) *t* = 1.38 ms; (d) *t* = 4.80 ms.

**Figure 11 fig11:**
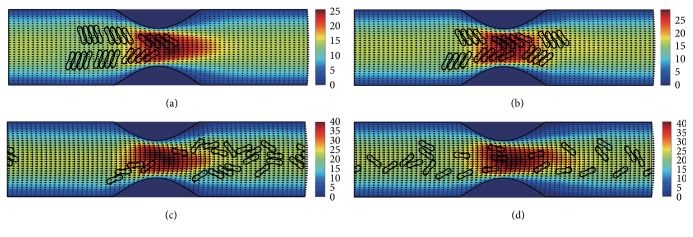
Motion of red blood cell aggregates (configuration in Figure 2(b)) in the microchannel with 50% stenosis at time instants (a) *t* = 0.37 ms; (b) *t* = 0.48 ms; (c) *t* = 1.34 ms; (d) *t* = 4.80 ms.

**Figure 12 fig12:**
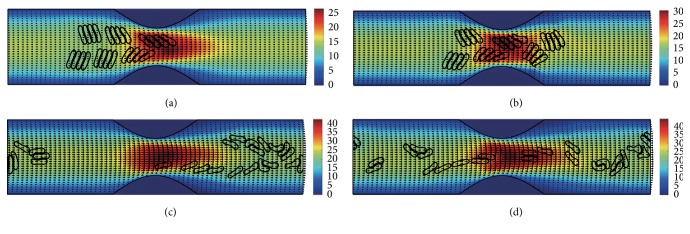
Motion of red blood cell aggregates (configuration in Figure 2(c)) in the microchannel with 50% stenosis at time instants (a) *t* = 0.37 ms; (b) *t* = 0.47 ms; (c) *t* = 1.37 ms; (d) *t* = 4.80 ms.

**Figure 13 fig13:**
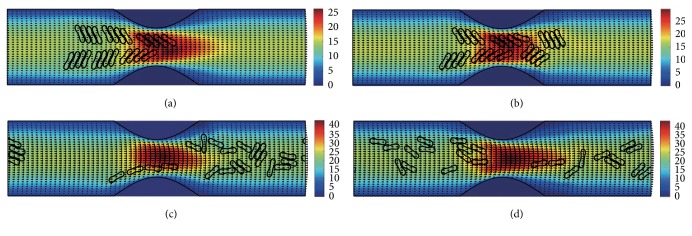
Motion of red blood cell aggregates (configuration in Figure 2(d)) in the microchannel with 50% stenosis at time instants (a) *t* = 0.37 ms; (b) *t* = 0.48 ms; (c) *t* = 1.37 ms; (d) *t* = 4.80 ms.

**Figure 14 fig14:**
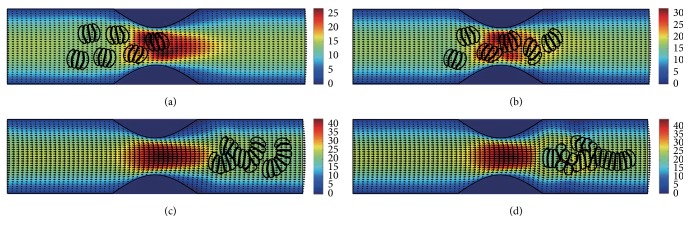
Motion of red blood cell aggregates (configuration in Figure 2(e)) in the microchannel with 50% stenosis at time instants (a) *t* = 0.37 ms; (b) *t* = 0.48 ms; (c) *t* = 1.36 ms; (d) *t* = 4.80 ms.

**Figure 15:**
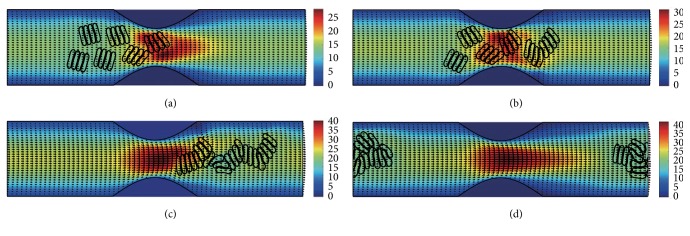
Motion of red blood cell aggregates (configuration in Figure 2(f)) in the microchannel with 50% stenosis at time instants (a) *t* = 0.37 ms; (b) *t* = 0.49 ms; (c) *t* = 1.35 ms; (d) *t* = 4.80 ms.

Figures 10 [Fig fig11] [Fig fig12] [Fig fig13] [Fig fig14]–15 show the motion of 6 RBC aggregates in the microvessel with 50% stenosis. The parameters and the initial configuration of the aggregates were the same as in the last section. It has been shown in the simulations that the velocity of the blood flow at the throat of the stenosis increased comparing to the 40% stenosis vessel. The disassociation of the aggregates and the deformation of the RBCs were similar to the results for the 40% stenosis when the aggregating strength was weak or mild. However, when the aggregating strength became strong, the larger agglomeration or rouleaux of RBCs were formed. The reason for this phenomenon is because that the increase of stenosis severity increased blood velocity at the stenosis. Thus, the downstream cells easily caught up the upstream cells which were slowed down by the friction of the vessel wall.

#### 3.2.3. In the Channel with 60% Stenosis

**Figure 16:**
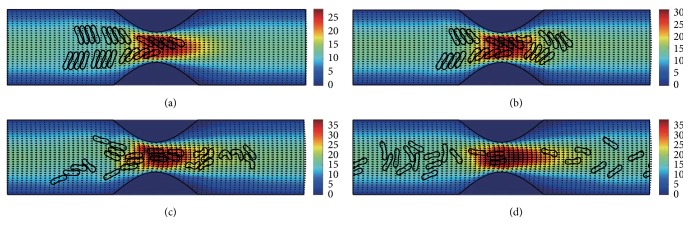
Motion of red blood cell aggregates (configuration in Figure 2(a)) in the microchannel with 60% stenosis at time instants (a) *t* = 0.39 ms; (b) *t* = 0.51 ms; (c) *t* = 1.37 ms; (d) *t* = 4.80 ms.

**Figure 17 fig17:**
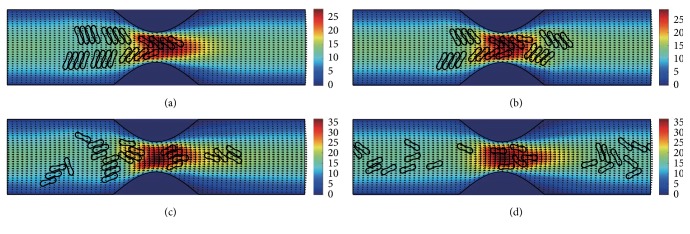
Motion of red blood cell aggregates (configuration in Figure 2(b)) in the microchannel with 50% stenosis at time instants (a) *t* = 0.39 ms; (b) *t* = 0.52 ms; (c) *t* = 1.37 ms; (d) *t* = 4.80 ms.

**Figure 18 fig18:**
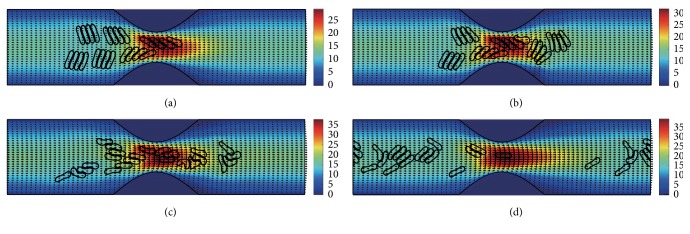
Motion of red blood cell aggregates (configuration in Figure 2(c)) in the microchannel with 50% stenosis at time instants (a) *t* = 0.39 ms; (b) *t* = 0.51 ms; (c) *t* = 1.35 ms; (d) *t* = 4.80 ms.

**Figure 19 fig19:**
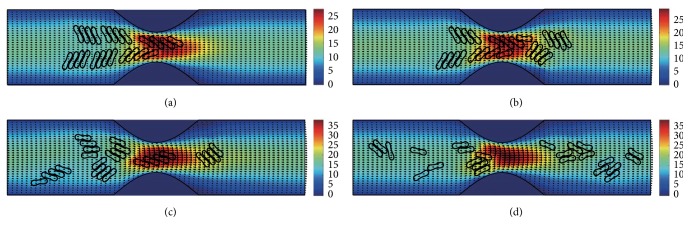
Motion of red blood cell aggregates (configuration in Figure 2(d)) in the microchannel with 50% stenosis at time instants (a) *t* = 0.39 ms; (b) *t* = 0.52 ms; (c) *t* = 1.34 ms; (d) *t* = 4.80 ms.

**Figure 20 fig20:**
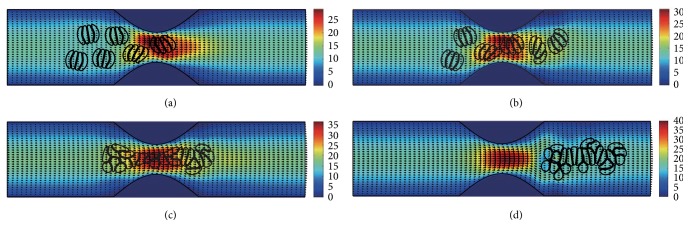
Motion of red blood cell aggregates (configuration in Figure 2(e)) in the microchannel with 50% stenosis at time instants (a) *t* = 0.39 ms; (b) *t* = 0.52 ms; (c) *t* = 1.38 ms; (d) *t* = 4.80 ms.

**Figure 21:**
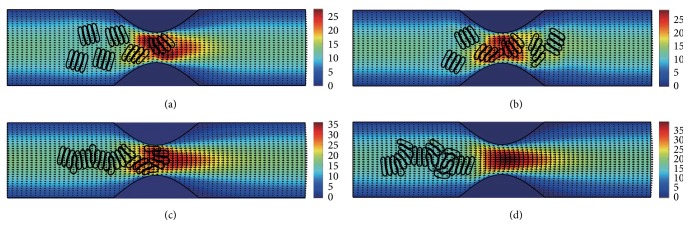
Motion of red blood cell aggregates (configuration in Figure 2(f)) in the microchannel with 50% stenosis at time instants (a) *t* = 0.40 ms; (b) *t* = 0.53 ms; (c) *t* = 1.36 ms; (d) *t* = 4.80 ms.

Simulation also has been done in the microchannel with 60% stenosis and the results are shown in Figures 16 [Fig fig17] [Fig fig18] [Fig fig19] [Fig fig20]–21. As the stenosis severity increased to 60%, the microvessel was blocked more than the last two cases. Unlike the cases of 40% and 50% stenosis, the aggregates with mild aggregating strength did not dissociate completely even when the elapsed time was long enough. On the other hand, they almost kept their original configuration for a long time (Figures 18(c) and 19(c)) before some of them disassociating into smaller aggregates or individual cells. Furthermore, aggregates formed by RBCs with strong aggregating strength attracted together and more compact aggregates were observed than in 50% stenosis case. It is reasonable to conclude that the increase of stenosis severity facilitated aggregation of malaria-infected RBCs. More disturbance of the flow at the throat of the stenosis was also observed.

In general, for all three stenosis levels, healthy RBCs and aggregates with ring stage or early trophozoite-stage infection (configurations in Figures 2(b)–2(d)) passed through the stenosis easily and dissociate into individual cells or smaller aggregates. They also recovered their normal biconcave shape quickly after passage through the constriction. However, configurations in Figures 2(e) and 2(f) maintained and even formed larger aggregates in the stenotic vessel. They tended to block the flow at the mouth of the stenosis and this can be seen from the decrease of the flow velocities in the straight section of the channel. Thus, the delivery of nutrients and removal of toxins by RBCs will be severely decreased and the phenomena show the behavior of late-stage trophozoite. These results are qualitatively consistent with the in vivo experimental findings [3].

### 3.3. Flow Characteristics

#### 3.3.1. Effect of Stenosis Magnitude

**Figure 22:**
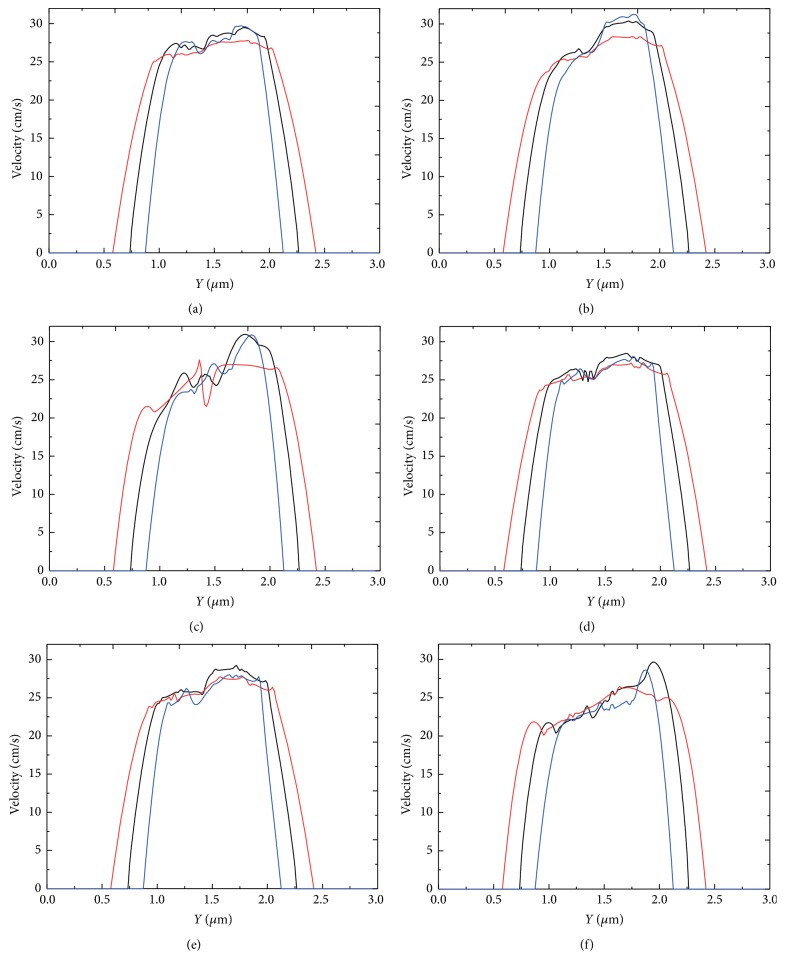
Blood velocity profile at the throat of the stenosis for three stenosis magnitudes: 40% stenosis (red lines); 50% stenosis (black lines); 60% stenosis (blue lines). (a) *D*_*e*_ = 0.01 *μ*J/m^2^ and *k*_*b*_ = 3.0 × 10^−13^ Nm; (b) *D*_*e*_ = 0.1 *μ*J/m^2^ and *k*_*b*_ = 3.0 × 10^−13^ Nm; (c) *D*_*e*_ = 1.0 *μ*J/m^2^ and *k*_*b*_ = 3.0 × 10^−13^ Nm; (d) *D*_*e*_ = 0.01 *μ*J/m^2^ and *k*_*b*_ = 3.0 × 10^−12^ Nm; (e) *D*_*e*_ = 0.1 *μ*J/m^2^ and *k*_*b*_ = 3.0 × 10^−12^ Nm; and (f) *D*_*e*_ = 1.0 *μ*J/m^2^ and *k*_*b*_ = 3.0 × 10^−12^ Nm.

In Figures 22(a)–22(f), velocity at the stenosis verses radial location for three aggregation forces and two cell membrane constants are shown in the same panel for three stenosis magnitudes. The comparison shows that the velocity increased as the stenosis increased. This trend was more profound for the soft cells with mild and strong aggregating strength. It can also be seen from Figure 22 that velocity profiles were more distorted when the aggregating strength was strong. The asymmetry of the velocity profiles was mainly due to the initial asymmetric location of the aggregates.

#### 3.3.2. Effect of Aggregation Force

**Figure 23:**
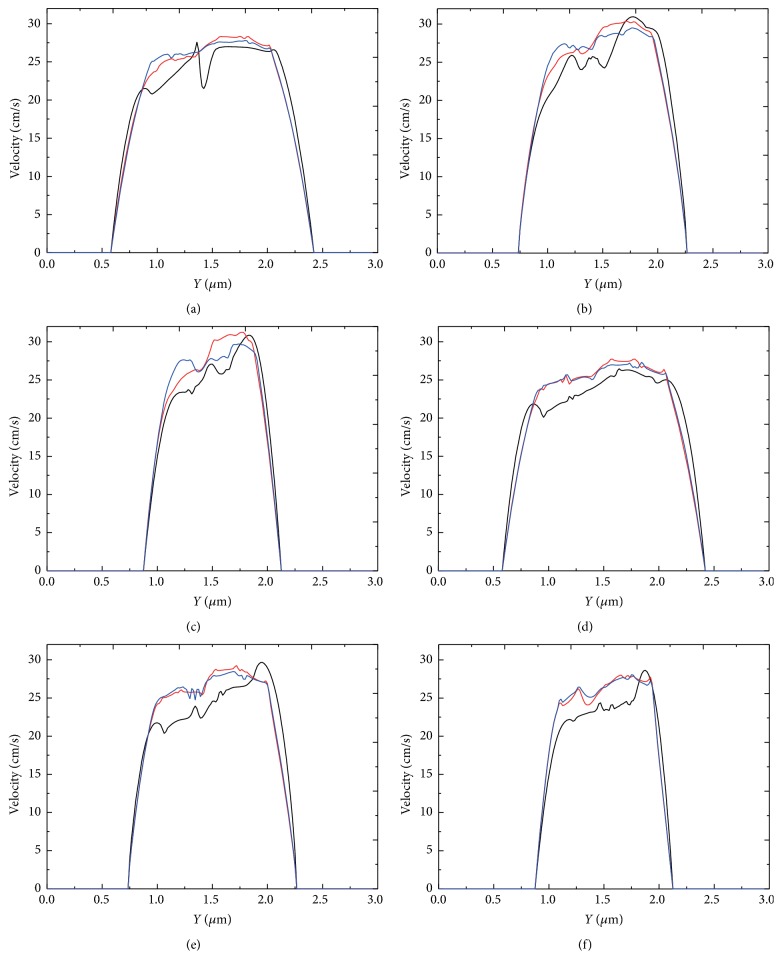
Blood velocity profile at the throat of the stenosis for three aggregating strengths: *D*_*e*_ = 0.01 *μ*J/m^2^ (red lines); *D*_*e*_ = 0.1 *μ*J/m^2^ (blue lines); *D*_*e*_ = 1.0 *μ*J/m^2^ (black lines). (a) *k*_*b*_ = 3.0 × 10^−13^ Nm, 40% stenosis; (b) *k*_*b*_ = 3.0 × 10^−13^ Nm, 50% stenosis; (c) *k*_*b*_ = 3.0 × 10^−13^ Nm, 60% stenosis; (d) *k*_*b*_ = 3.0 × 10^−12^ Nm, 40% stenosis; (e) *k*_*b*_ = 3.0 × 10^−12^ Nm, 50% stenosis; and (f) *k*_*b*_ = 3.0 × 10^−12^ Nm, 60% stenosis.

Next we investigated the effect of aggregation force on the velocity profile at the throat of stenosis (Figure 23). It is noted that the flow velocity at the throat of the stenosis decreased as the aggregation force increased. This trend was found for all the stenosis magnitudes and both the soft and the rigid red blood cells. However, it is also observed that the decrease of the velocity was not linear. When the aggregation force increased from weak (blue lines) to mild (red lines), the results showed little to no effect on the velocity. While when the aggregation force became strong (black lines), the effect was significant. The results indicated that increasing of aggregation strength up to a certain level leaded to increase of flow resistance in the stenotic vessels.

#### 3.3.3. Effect of Cell Deformability

**Figure 24:**
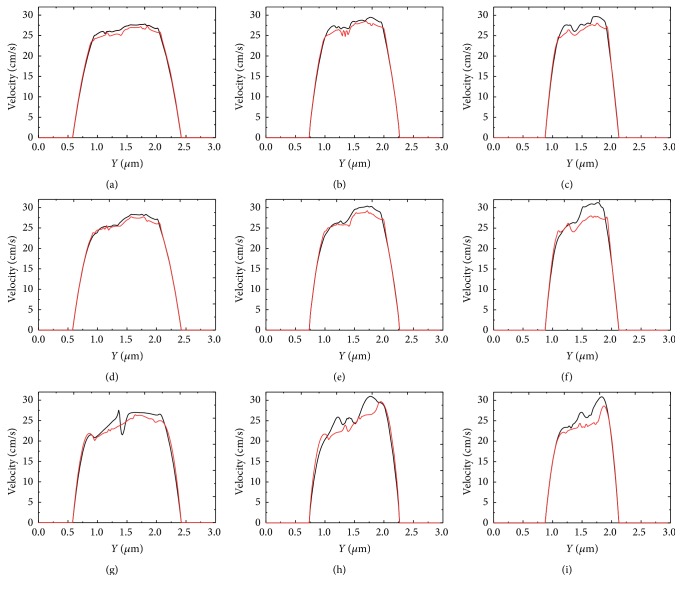
Blood velocity profile at the throat of the stenosis for two values of RBC deformability: *k*_*b*_ = 3.0 × 10^−13^ Nm (red lines); *k*_*b*_ = 3.0 × 10^−12^ Nm (black lines). (a) *D*_*e*_ = 0.01 *μ*J/m^2^, 40% stenosis; (b) *D*_*e*_ = 0.01 *μ*J/m^2^, 50% stenosis; (c) *D*_*e*_ = 0.01 *μ*J/m^2^, 60% stenosis; (d) *D*_*e*_ = 0.1 *μ*J/m^2^, 40% stenosis; (e) *D*_*e*_ = 0.1 *μ*J/m^2^, 50% stenosis; (f) *D*_*e*_ = 0.1 *μ*J/m^2^, 60% stenosis; (g) *D*_*e*_ = 1.0 *μ*J/m^2^, 40% stenosis; (h) *D*_*e*_ = 1.0 *μ*J/m^2^, 50% stenosis; and (i) *D*_*e*_ = 1.0 *μ*J/m^2^, 60% stenosis.

The cell membrane deformability also had an effect on the velocity of blood flow at the stenosis and the results are revealed in Figures 24(a)–24(i). Two membrane constant values have been used in the simulations and two types of RBCs were modeled with these constants, namely, soft cells and rigid cells. In Figure 24, black lines represented the velocity profile at the throat of the stenosis of the blood flow with soft RBCs, and red lines are for the blood flow with rigid cells. Overall, the flow velocity decreased with the increase in stiffness of the cell. In particular, the effect was more profound when the stenosis was more severe or when the aggregation force was stronger.  Figure 24(a) shows the velocity profile for the case of 40% stenosis and the weak aggregation force. The velocity profile was relatively smooth and less disturbance was observed on the curve. On the contrary, the velocity profiles were rougher in narrower stenotic vessels or when the aggregation of the RBCs was more severe.

## 4. Conclusions

The main objective of this work is to probe how changes in the cell membrane rigidity, the aggregating strength, and the magnitude of the stenosis affect the transit of the malaria-infected RBC aggregates through stenotic microchannels. Two types of RBCs, namely, soft and rigid, have been studied under three different levels of aggregating strength. The simulations were performed over a range of stenosis magnitudes: 40%, 50%, and 60%.

It has been found in this study that soft RBCs with strong aggregating force formed the most compact aggregates. In the aggregates, the RBCs experienced large deformation. Rigid ones with weak aggregating force formed the loosest aggregates. In these aggregates, the RBCs maintained their normal biconcave shape. In blood flow, the aggregates of weak aggregating strength were more likely to dissociate to individual cells, while the aggregates with strong aggregating strength would form even larger aggregates or rouleaux. The rigidity of the cell membrane hindered passage of the cell through microchannels and this result is qualitatively in agreement with experimental findings on the rheological behaviors of malaria-infected RBCs through a narrow constriction in a microchannel. The flow velocity at the throat of the stenosis decreased with increasing membrane modulus and more deviation from the parabolic profile was observed, especially for the narrower stenosis and stronger aggregating force.

The present study used a two-dimensional model to qualitatively simulate malaria-infected RBCs in stenotic microvessels. It is important to extend this algorithm to the physiologically relevant hematocrit contents in three-dimensional situation and compare the results with experimental observations quantitatively. It is also quite interesting to adapt the model for the more thorough investigation of RBC rheology at various stages of malaria infection as well as its impact on leukocyte migration. In addition, it provides a potential approach to investigate drug delivery at cellular level in microvessels involving infected RBCs.

## Figures and Tables

**Table 1:** Parameters used for the simulations.

Parameter	Symbol	Value
Blood plasma density	*ρ*	1.0 g/cm^3^
Blood plasma viscosity	*μ*	1.2 cp
Aggregating energy constant	*D*_*e*_	0.01, 0.1, 1.0 *μ*J/m ^2^
Scaling factor	*β*	80^ ^ *μ*m^−1^
Reference length	*r*_0_	0.49^ ^ *μ*m
Spring constants for red blood cell membrane	*k*_*b*_	3.0 × 10^−13^, 3.0 × 10^−12 ^Nm
Length of the channel	*L*	140^ ^ *μ*m
Radius of inlet (outlet)	*R*	30^ ^ *μ*m
Grid size	*h*	1/64^ ^ *μ*m
Time step	Δ*t*	1 × 10^−5 ^ms^−1^
